# On Spontaneous Hæmorrhage

**Published:** 1816-06

**Authors:** Walter Channing


*For the London Medical and Physical Journal.*

### *On Spontaneous Hæmorrhage;* by Walter Channing, M.D.

A NUMBER of cases of spontaneous hæmorrhage having occurred in the writer's practice, he was led to make the following inquiries and reflections on their probable sources and proximate cause. The cases consisted of hæmorrhage from the bladder in mild fever in one subject. From the rectum, in two cases. In these cases the loss of blood was very considerable, without any previous affection of the organ that was apparent, and in which a slight derangement only in some of the functions of the body had occurred a very few hours before the hæmorrhage. In three cases the hæmorrhage occurred from the calf of the leg. Of these, two were adult females, the third an adult male. In neither of them were the veins varicose, and in only one was there any ulceration, and in this the ulcerated surface was not much larger than that of a split pea. In one case the hæmorrhage occurred from the lungs under somewhat peculiar circumstances; one was a case of scurvy, and a number of them cases of epistaxis during measles. If there should be nothing new in any of the following inquiries and reflections, to any reader of the Journal, a principal object in their publication will be answered, if they lead to more elaborate and accurate investigations of the subject. The first inquiry is into the most probable sources of spontaneous hæmorrhages.

Among the many opinions which have been held on this point, two only will be mentioned. The first supposes the blood blood to flow from the mouths of those vessels which open into cavities, into secreting organs, into the receptacles of secretion, and into muscular and other structures; the great agents of supply and other important functions, constituting the exhalent and secretory systems. This doctrine of the sources of hæmorrhage has been designated by the term *anastomosis*. The second is termed *diapedesis* or *transudation*. This teaches that hæmorrhages proceed from pores with which the body is every where replete, and from those interstices which it is supposed may be formed, by accident or disease, by a greater than natural separation of the ultimate particles of which the solids are made, or, supposing their natural state to exist, in consequence of a greater than natural fluidity of the blood.

The first of these opinions appears most consonant with what we best know of the human body. For, though disease may be regarded, in some measure, as an accidental occurrence or state of the system, it generally appears to us so modified by, or under the influence of what is natural or peculiar to the living body, as to permit, in some considerable degree, the usual phenomena of health; and, although occasionally the natural functions of an organ may apparently cease in consequence of the violence of disease, still the diseased actions go on in the same vessels, in which the healthy ones resided; thus making these last ultimately the means, agents, or seats of curative processes. Farther, while we regard the various actions of the healthy body as the ultimate object of certain structures, we not only feel unwilling, but perceive no necessity to exist, to resort to other modes of explaining disease. While we retain what may be called at least a natural physiology, we seem in our investigation of disease to pursue a rational pathology.

The strongest arguments, however, for this opinion, are not drawn from its reasonableness; others more satisfactory may be derived from the condition of the general system, that of parts only, and peculiarities of the organs themselves under which hæmorrhages most commonly occur.

The greater number of cases which are mentioned in the beginning of this paper, are of hæmorrhage, which occurred among the other phenomena of disease. Some of them were unusual occurrences, others among the more usual symptoms of morbid action. The most striking were those hæmorrhages which took place from the bladder and lungs. The circumstances under which that occurred from the bladder were peculiar. The subject, an extremely delicate child, had been sick for some days, was extremely reduced by disease; an unusual flow of urine took place, which was soon followed by blood from the urinary organ. This case goes far in support of the opinion adopted in this paper. The general state of the system predisposed the organs in general to take on diseased action. The functions of the kidneys became principally disturbed. So greatly was their secreting power increased in the first place, as to produce a very unusual quantity of urine. The condition, whatever it was, of the vessels or the organs subsequent to this action, permitted the escape of the blood along with the natural secretion. The affection, most probably, did not amount to inflammation; hence no pain was complained of, or it was so low a degree of inflammation as only to be followed, first, by increased urine and then blood.

Farther, the rare occurrence of hæmorrhage from organs labouring more or less under disease, is not an argument against the doctrine of anastomosis; but, were the notion of transudation true, we might be rationally surprised that we do not more often find effusion of blood into the various cavities of the body. In this last case the effusion would be merely an accidental, not an unusual occurrence. In the first, on the contrary, it can only occur as a consequence of the living agency, and be among the anomalous occurrences of diseased function.

If the remarks just made be true, their application to the more common cases of hæmorrhages may be extended without violence, to those cases also to which the doctrine of transudation would appear to have a more legitimate reference. I now refer particularly to those which appear under circumstances of peculiar malignity, which, from their phenomena, have received the appellation of putrid; and in which, not only the solids, but the circulating fluids have undergone certain changes, in consequence of which some of their distinctive living characters, and more especially those of the last, appear in a measure lost.

But, if blood of its usual qualities can and does at times pass extremely minute vessels, vessels which have very peculiar offices in the system to perform, and originally were never designed to give passage to the blood, *a fortiori*, how much more readily will the blood find a passage through the same vessels, attenuated as it is by disease, and the vessels themselves, in their want of function or action presenting but a slight obstacle to its escape?

Again, it has been asserted by those who support the opinion of transudation, that were hæmorrhage truly the consequence of anastomosis, we should very often find it occur in those higher actions of which those vessels are susceptible and so often suffer; for instance, during a state of inflammation.

tion. And the argument is supported by the increased secretion which inflamed organs at times afford.

Now, it is a well known fact, that inflammation is at times attended with increased secretion, and sometimes even by effusions of blood from the affected organ. But, as inflammation is a process totally unlike the natural, or rather healthy, functions of a part or organ, attended by its very nature with very extraordinary, but fixed, peculiar, I might say natural, phenomena, we should almost, nay quite, as soon, look for hæmorrhage during the most healthy state and functions of an organ, as during the occurrences of genuine inflammation. During genuine inflammation, the vessels are called upon to do something more than in health; they are not merely to preserve the integrity of the organ to a degree consistent with its vitality, but in taking on themselves new functions, to restore to it health. All that is designed in these remarks, is to show that, because the secretions of an organ may be increased by inflammation, or what appears to be genuine inflammation, and because hæmorrhage is not always among its phenomena, we are not hence to infer that its sources are not vessels, and its occurrence not among the actions of which those vessels are susceptible.

If we advert for a moment to diseases in which hæmorrhages are most frequently found to occur, we shall find them diseases of organs remarkably vascular, and perpetually performing very important secretory functions. Thus we find the diseases of the mucous membranes most frequently accompanied with hæmorrhages, and next to these, those of the serous membranes.

Dysentery, that distressing affection of the mucous membrane of the lower portion of the intestinal canal, furnishes us with a striking illustration of these remarks. All the facts connected with its peculiar phenomena, viz. the discharges which characterize it, lead us to the vascular origin of its occasionally accompanying hæmorrhage. We have, for instance, in the first place, the increased mucus covering the fæces; then pure mucus, then mucus slightly tinged with blood, and at last the simple coagulum of blood itself, or that coagulum combined with portions of lymph which the motion of the intestines, their pressure or other causes have detached from the common mass, or which the small vessels have immediately yielded; and sometimes a small portion of mucus, that the lowest vessels of the rectum may be still able to secrete.

When we examine the body after death from this disease, we discover, not an abraded surface of mucous membrane covered with pus, but a surface smeared with a bloody mucus, or covered with a layer of lymph, similar to the discharges charges during life; and when we have removed these, we sometimes observe the turgid secreting vessels, constituting those dots which authors have mentioned; at other times other appearances of inflammation; while sometimes we only observe marks of organic disease so slight, as to be scarcely visible. The phenomena just particularized may all be satisfactorily explained without referring to any of them, either to the abrasion to which an ulcerative process might be liable under the existing circumstances of the disease, or to that partial disorganization which the violence of the disease might be conjectured to induce, and which has been considered by some so favourable for hæmorrhage by transudation.

If spontaneous hæmorrhages have their source in vessels, the next subject for inquiry is their proximate cause, or that state of vessels of which they are the effect. To answer this question, there are far greater difficulties to surmount, than were met with in pursuing the first inquiry, and, at last, we can arrive at but a rational hypothesis. In the greater hæmorrhages which occur from secreting organs, properly so called, we find in the first place a partial temporary suspension of the secreting function, though, at times, the hæmorrhage is preceded by an evident increase of the secretion. The vessel, in other cases, which heretofore delayed the blood, (if I may so say), that it might select from that fluid what was most appropriate to the performance of its function in the economy, or that it might from the whole mass elaborate its secretion, (which, by its healthy structure and during its healthy functions, could not admit the passage of the fluid in its entire state,) is now found to give the blood a free course. The peculiar irritability with which the vessel was endowed, and which preserved, while it constituted, its healthy tone, is found to a degree wanting, and the stimulus of what may be called a foreign body is not perceived by it. If we examine the purely exhalent system, from which, at times, hæmorrhages proceed, we also find, that that has undergone great changes from its natural state and function. Instead of separating from the common circulating mass, that portion of it which will answer its peculiar purposes in the economy, and the just quantity of which is so exactly preserved by the balance between exhalation and absorption, we find the blood itself effused; at times, in such quantities, merely as to produce but slight inconvenience, at others, fatal effects; its consequences always depending on the circumstances inducing or attending the discharge, and on the cavity into which it is made. Let us now inquire, whether the state of the vessels, favourable to hæmorrhage, be secondary to a peculiar condition of the general system—in fact, be produced by it, and simply a symptomatic affection? or is hæmorrhage the consequence or symptom of a primary, idiopathic affection of the small vessels, of which the peculiar state of the circulation, and the morbid condition of the whole system are but symptomatic?

This inquiry is, perhaps, of more importance than at the first sight it may appear. Hæmorrhage is certainly among the alarming phenomena of disease, and at times, and with the uninformed always, among the most alarming that occur. It is, however, a fact, that it is by no means always among the most dangerous phenomena of disease, and at times it appears to us under the character, and certainly with the effect of a curative process.

To the above questions, it will be perfectly safe and perfectly true to answer, that hæmorrhage undoubtedly occurs as a consequence of an original general disturbance in the system, and is thus among its symptoms; and is also, in other cases, the effect of an original affection of those vessels from which it proceeds. The vessels from which it may proceed, are remarkably under the influence of the various causes which affect the body, and particularly the more powerful ones, and especially during much local or general debility. Thus we find them, at times, influenced by the state of the mind. A patient, ill of pulmonary disease, requested a consultation. In this case, hæmoptysis had never occurred. The disease, though of long standing, had not apparently produced great disorder in the lungs. It seemed chiefly confined to the mucous membrane. She was a little agitated by the presence of her new physician, which agitation, however, soon subsided, and as she was coughing, as she usually did, the physician begged her to permit him to see the matter expectorated. It was found to be chiefly blood. It produced the alarm and distress that such an occurrence might be supposed to excite; they soon subsided, and in a few hours, with very simple remedies, the hæmorrhage ceased, and has not again appeared. The disease has not essentially altered. In this case a very sudden change was effected in the state and functions of the small vessels. It was one of the ultimate effects of some operation of the mind. The small vessels were suffering a debility induced by protracted, though slight organic disease of the lungs, and in which they were most intimately concerned. The circulating system also possessing a morbid mobility, was suddenly affected by the mind. An unusual quantity of blood was thrown on the small vessels, and they readily gave it a passage through and from them. In this case, although the hæmorrhage morrhage depended on the altered state of the circulating system as its exciting cause, it was, nevertheless, in part, the effect of the previous condition of the small vessels, viz. of their predisposing debility. A case is related in the Parisian Journal of Medicine for January, 1815, edited by Sédillot, in which the hæmorrhage occurred from the skin; the blood freely passing from the vessels of perspiration. This remarkable occurrence, was among the phenomena of *nephritic colic*, which disease was directly and immediately produced by a violent fit of anger. The small vessels appear, in the case of this woman, to have been remarkably susceptible of the action on which hæmorrhages depend, and which was produced by moral causes. During a fit of anger, two years previous to the bloody perspiration, considerable hæmoptysis and cough occurred; and this was always the consequence of mental disturbance, unless peculiar nervous symptoms, to which she was also liable, were produced by the fit of anger. The *last* paroxysm of the colic she suffered, took place without manifest cause; and the hæmorrhage was, in this instance, confined to the skin of the face, neck, arm-pits, and anterior part of the thorax and abdomen. At the first view of this case, the bloody perspiration which occurred among its phenomena, may be considered a sympathetic affection, the original one being the nephritic colic. With more correctness, however, may it not be considered as the direct consequence of that state of mind which induced the colic; and this, notwithstanding its occurrence during a paroxysm of colic, which was not the consequence of the original cause, viz. the mental disturbance? The hæmorrhage had occurred repeatedly with the colic, and thus they had become intimately associated, not as cause and effect however, but as the effects of any cause which might be sufficient to the production of either of them. The small vessels of the skin and those of certain abdominal viscera, were precisely in the same state of predisposition to any morbid impression. The varied and remarkable effects of such impression, however, depended on circumstances peculiarly calculated to admit of their production. The hæmorrhages, in these cases, were the consequence of derangement in the whole system, were in short secondary affections in the small vessels. In leaving these, to consider those which proceed directly from the morbid actions of the small vessels themselves, we enter the most interesting and widely extended field of pathology.

What very important morbid action, what disease can we advert to, in which the small vessels are not most intimately concerned? Dysentery, which we have before adverted, what is it but a disturbance in the functions of the small vessels sels of the intestinal tube, most intimately associated with a similar one of those of the skin? It is true, that in the milder cases of this disease, hæmorrhage may not appear. This is very easily explained, without yielding the position that they are the seats of hæmorrhage when that does occur. They are able, by their great and easy adaptation to new occurrences in the system, to dispose of, or relieve themselves of the great afflux of blood to them, by exerting themselves in an extraordinary degree. But when the impression of the exciting causes of the disease has been very violent, when especially the predisposition has been perfectly formed; after a few days of violent secretion, of violent effort to effect a cure, if I may so say, their natural healthy functions in a great degree cease, and pure blood passes unimpeded into the intestinal canal. In this case, then, hæmorrhage legitimately belongs to the small vessels as the immediate effect of their own actions. If these are not soon suspended by those means which diminish morbid irritability in them, as in all other organs of similar properties, they communicate to the system at large, or excite in it, and directly too, disturbances which advance with more or less rapidity to a fatal termination. I might mention here the fatal convulsions which have occurred in dysentery, in children, this season at this place.

Take another disease, an inflammation, for instance, of the lungs. In this affection, notwithstanding the general disturbance which exists in the system, the rapid and strong pulsation of the heart and great vessels, the heat, &c. which might lead us to trace the local affection entirely to that of the general system, the small vessels of the diseased organ, may perhaps more correctly be considered as primarily affected, and as suffering disease from their intimate association with those of the surface; and in the advanced stages of the disease, when hæmorrhage becomes its accompanying phenomenon, they are not only the great agents of the hæmorrhage, but to their diseased functions are to be then traced the various morbid affections which are exhibited in the general system. That this reasoning is true, we have only to advert to the treatment of this disease, which consists of two great objects, viz. to diminish the action of the small vessels; and then to induce new actions, which on the principle of mere change effects the cure; or, by suspending disease, leaves the organ at liberty to resume its healthy operations.

There is nothing more common in our pathological observations, than to view diseases in the relations of cause and effect. It has been an object in this paper to consider diseases, which appear to have these relations, not exactly in this light, but to argue that the predisposition to disease in

 any any one organ may not have been confined to that alone, but has been extended to other organs in its neighbourhood; and that the operation of the exciting causes on each, in the production of the disease, has appeared sooner or later in one or the other, in consequence of peculiar circumstances of structure or function, or of susceptibility to the action of those exciting causes; not in consequence of the mere operation of sympathy.* To illustrate this reasoning, I would advert to some very remarkable cases of hæmorrhage constituted by a vicarious performance of a certain uterine function, viz. the catamenia. Thus the catamenia, instead of appearing from the cavity of the uterus, have been secreted into the cavity of the abdomen; or, in place of uterine hæmorrhage at the proper period, hæmorrhage has occurred from the serous vessels of the peritoneum. Now, in this case, the predisposing causes of obstruction affected the small vessels in the neighbourhood of the uterus; and the exciting causes found the first more susceptible of their influence than those of the last, or, what is more probable, the exciting causes of the obstructions directly affected the small vessels; and the derangement of the general system was more readily relieved through them, than through the secreting function of the uterus.

These remarks have of course a limited extent. They are meant to support the opinion of the primary affection of the small vessels in the species of hæmorrhage under consideration. It would be interesting to extend these remarks to various other species of spontaneous hæmorrhage. Scurvy offers us an instance in which this phenomenon is very generally to be observed. The proximate cause of hæmorrhage in this case is strictly an idiopathic affection of the small vessels. The peculiar condition of these vessels on which the hæmorrhagic action depends is very extensive in this disease. For, though we find the actual escape of blood from the body occurring sensibly from the gums, we also find a strong tendency to the same phenomenon in every part of the body which comes under our notice. We observe it particularly in the skin. Now, although there be much in the cuticular spots, which are observed in this disease, which

* It may be replied here, that even under these circumstances of extended and universal predisposition, the fact of a progression in diseases proves the doctrine of sympathy. All that is meant in the above remark is, that the second organ is not affected with disease merely in consequence of the disease of the first, but that the order of occurrence is the consequence of circumstances altogether foreign from sympathy.

resembles resembles some of the exanthemata both in their access, progress, and decline, perhaps they would with more correctness be said to owe their irregular appearance to the various degrees of predisposition the small vessels possess, to an effusion into the skin; that in short, such an universal debility in them as would admit an universal and simultaneous escape of blood into the skin, and cellular structures, would amount to positive death. We are to consider the gums and small vessels of the skin, in common with the whole system, under the influence of the predisposing and exciting causes of scurvy, that the hæmorrhage in fact is but a symptom of the idiopathic affection of these vessels.

A classification of hæmorrhages has suggested itself in the course of the preceding inquiries, which, it was thought, would render their investigation more easy, and the communication of what might be discovered, far more perfect, than that which is founded on their remote or exciting causes. This latter method has been adopted in the divisions of M. Lordat, who published a work a few years ago, expressly on the subject of hæmorrhages. The classification suggested, is to be founded on the *textures*, or organs from which most generally the hæmorrhages proceed. Thus hæmorrhages from the mucous membrane should constitute one class or genus; those from the serous another; the muscular organs a third, &c. &c. while the species should be founded on prominent circumstances more immediately concerned in the actual flow of blood.

The writer is aware that this method is condemned by M. Lordat. But this judgment of this highly respectable author, he is also aware, was the consequence of the peculiar notions of that author, concerning the share the small vessels of the various textures or organs of the living body have, in the production of hæmorrhage, viz. that they are but secondarily concerned in its production.

Something may have been expected in the course of this paper relative to the modes of treatment of hæmorrhages. The limits prescribed by the nature of the work in which the paper appears, have prevented an investigation into the modes of treatment. In fact, the treatment of hæmorrhages resolves itself either into the treatment of those diseases in which they appear, or which they constitute; or, when it is but a purely symptomatic and sudden affection, the consequence of derangement of the whole system, into the treatment, or means of relief of the general system, with whose disorder it is associated, or of which it is but a symptom.

Hæmorrhages, as they have been considered in the preceding pages, are very often the results of disease in the

 small small vessels. These vessels are intimately connected with the whole circulating system. This last, of course, is always more or less affected by the diseases under notice, and in a majority of their diseases it is believed that the best means of cure may be found in direct depletion from the circulating system. The writer has seen with pleasure the practice of bleeding becoming more and more extensive in this part of the country; and on unquestionable authority would mention the success of bleeding in some cases of our dysenteries.

Hæmorrhages on the other hand depending on other circumstances will require their appropriate treatment. Those which are produced by sudden commotions of mind or body, or both, will at times be best treated by bleeding, at others by narcotics or antispasmodics; others the consequence of peculiar courses of diet will be best treated by entire change in the food, and by tonics. Scurvy furnishes us with instances of this kind; while critical discharges of blood will cease with the other circumstances attending crisis. Febrile diseases attended with petechiæ, and discharge of blood from various organs, of course, will demand appropriate treatment.

The question of the precise nature of the action of the small vessels, on which hæmorrhage depends, although involved in the preceding inquiries, has been carefully avoided. The motives for this silence, the writer confesses to have been others, and those more powerful, than the length to which the paper has already extended. This last motive has however had some influence with him in bringing it to a close.—*New-England Journal.*

